# Incorporating genome-wide and transcriptome-wide association studies to identify genetic elements of longissimus dorsi muscle in Huaxi cattle

**DOI:** 10.3389/fgene.2022.982433

**Published:** 2023-01-06

**Authors:** Mang Liang, Bingxing An, Tianyu Deng, Lili Du, Keanning Li, Sheng Cao, Yueying Du, Lingyang Xu, Lupei Zhang, Xue Gao, Yang Cao, Yuming Zhao, Junya Li, Huijiang Gao

**Affiliations:** ^1^ Institute of Animal Science, Chinese Academy of Agricultural Sciences, Beijing, China; ^2^ Jilin Academy of Agricultural Sciences, Changchun, China

**Keywords:** longissimus dorsi muscle, GWAS, TWAS, FCT, Huaxi cattle

## Abstract

Locating the genetic variation of important livestock and poultry economic traits is essential for genetic improvement in breeding programs. Identifying the candidate genes for the productive ability of Huaxi cattle was one crucial element for practical breeding. Based on the genotype and phenotype data of 1,478 individuals and the RNA-seq data of 120 individuals contained in 1,478 individuals, we implemented genome-wide association studies (GWAS), transcriptome-wide association studies (TWAS), and Fisher’s combined test (FCT) to identify the candidate genes for the carcass trait, the weight of longissimus dorsi muscle (LDM). The results indicated that GWAS, TWAS, and FCT identified seven candidate genes for LDM altogether: *PENK* was located by GWAS and FCT, *PPAT* was located by TWAS and FCT, and *XKR4*, *MTMR3*, *FGFRL1*, *DHRS4*, and *LAP3* were only located by one of the methods. After functional analysis of these candidate genes and referring to the reported studies, we found that they were mainly functional in the progress of the development of the body and the growth of muscle cells. Combining advanced breeding techniques such as gene editing with our study will significantly accelerate the genetic improvement for the future breeding of Huaxi cattle.

## Introduction

In ancient China, cattle, as the primary means of production, were mainly used as the draft ox and rarely considered the source of meat. With the rapid development of the economy, consumers’ demand for beef, concerning quantity and quality, has increased in China. There is an urgent need to improve the productivity and quality of beef for the beef breed in China by directly changing the production capacity of beef cattle.

Locating the genetic variation of important livestock and poultry economic traits is still essential for genetic improvement. The genome-wide association study (GWAS) has successfully identified thousands of loci associated with complex features (Watanabe et al., 2019). However, 90% of the associated single nucleotide polymorphisms (SNPs) are located in the non-coding region of the gene, and their functions still are unknown, so the molecular mechanism of phenotypic variation cannot be explained clearly (Cannon and Mohlke, 2018). Previous studies have proved that gene expression is important in the phenotype of human diseases (He et al., 2013), and many genetic variations associated with phenotypes were likely to be expression quantitative trait loci (eQTL) (Nicolae et al., 2010). Furthermore, eQTL can be used to estimate the effects on gene expression and then be combined with physical phenotypes to conduct transcriptome-wide association studies (TWAS) to identify pivotal expression–trait associations (Gusev et al., 2016). The TWAS algorithm has been successfully implemented to identify the causal genes for the essential quantitative trait in cattle (Koupaie et al., 2019; Liu et al., 2021).

In this study, we utilized three strategies to identify the candidate genes that significantly affect the producibility of Huaxi cattle. First, we applied GWAS to identify the candidate gene by using 1,478 Huaxi cattle genotypes with the phenotypes of longissimus dorsi muscle (LDM) weight. Second, we implemented TWAS with genotypes (1,478 individuals), gene expression data of 120 individuals (contained in the 1,478 individuals), and phenotypes. Third, we utilized an ensemble approach, Fisher’s test (Yu et al., 2008; Kremling et al., 2019), combining the results of GWAS and TWAS to identify the candidate gene. Finally, we analyzed the function and preliminarily explored the molecular mechanism of the candidate genes with Gene Ontology (GO) and Kyoto Encyclopedia of Genes and Genomes (KEGG) analyses, which was helpful to the following breeding of Huaxi cattle.

## Materials and methods

Animal resources and phenotype: The Huaxi cattle population, including 1,478 cattle born between 2008 and 2021, was established in Ulgai, Xilingol League, and Inner Mongolia of China. After weaning, all calves were moved to the Jinweifuren fattening farm in Beijing, where they shared uniform management and standardized feeding [they were fed with the total mixed ratio (TMR) according to the eighth revised edition of the Nutrition Requirements of Beef Cattle (NRC, 2006)]. Animals were slaughtered at 22–26 months of age with electrical stunning, followed by bloodletting. The weight of the longissimus dorsi muscle (LCM, kg) was weighed after being chilled at 4°C for 24 h.

Genotype and quality control: Genomic DNA was isolated from blood samples using the TIANamp Blood DNA Kit (Tiangen Biotech Co., Ltd., Beijing, China). DNA quality was acceptable when the A260/A280 ratio was in the range of 1.8–2.0. All individuals were genotyped using an Illumina BovineHD BeadChip that contained 770,000 SNPs. Quality control (QC) procedures were carried out using PLINK v1.9 (Purcell et al., 2007) to filter out SNPs with call rate <90%, minor allele frequency (MAF) < 0.05, and a significant deviation from the Hardy–Weinberg equilibrium (*p* < 10^−6^), and >10% animals with missing genotype data were removed from the analysis. Finally, 1,478 cattle with 607,198 SNPs on 29 autosomal chromosomes with an average distance of 3 kb were included in subsequent analyses.

RNA extraction, library construction, sequencing, and quality control: Total RNA was extracted from SAT samples using TRIzol reagent (Invitrogen, Life Technologies) following the manufacturers’ instructions. The RNA concentration, purity, and integrity were, respectively, analyzed on Qubit RNA Assay Kit (Life Technologies, CA, United States), NanoPhotometer Spectrophotometer (Thermo Fisher Scientific, MA, United States), and RNA Nano 6000 Assay Kit of the Bioanalyzer 2,100 system (Agilent Technologies, CA, United States). The high-quality samples with 28S/18S > 1.8 and OD 260/280 ratio >1.9 were applied for constructing cDNA libraries according to the protocol of IlluminaTruSeqTM RNA Kit (Illumina, United States). Samples that presented an RNA integrity number greater than 7.0 were then sent for paired-end RNA sequencing (read length 150 bp) on the Illumina NovaSeq 6,000 platform (Modi et al., 2021). The RNA sequencing was completed by Beijing Novogene Technology Co., Ltd. Trimmomatic (v0.39) was applied to remove the reads containing low-quality reads, poly-N, and adaptor sequences (Bolger et al., 2014). Sequentially, the clean reads were aligned to the *Bos taurus* reference genome ARS-UCD1.2 using HISAT2 (v2.2.1) (Lachmann et al., 2020), and then the generated SAM files were converted to BAM files through SAMtools (v1.11). featureCounts (v1.5.2) was used to estimate read counts (Liao et al., 2014).

GWAS: GWAS analysis of LDM traits based on the linear mixed model (LMM) was completed using GEMMA (Zhou and Stephens, 2012):

y=Xb+Sg+Zα+e,

where y is the vector of phenotypes, b is the vector of fixed effect including age, sex, farm, and the days of fattening, S is the indicator variables of SNPs (0, 1, 2), g is the effect vector of SNPs, α is the polygenic effect vector, $\alpha ∼N\left(0,K\sigma_{g}^{2}\right)$ , e is the random residual, and $e∼N\left(0,I\sigma_{e}^{2}\right)$ . In GWAS, the Wald test was used to test the SNP significance, and the threshold of the *p-*value was set at 1/m, where m is the number of SNPs (Wu et al., 2014).

TWAS: REML (restricted maximum likelihood) was utilized to evaluate the heritability of each gene base on the gene expression and cis-SNPs located within 1 Mb of the physical position of the gene. Then, the gene with significantly non-zero heritability will be incorporated in the subsequent analysis. For the preselected gene, Bayesian Sparse LMM (BSLMM) was used to estimate the effect values of the cis-SNPs for gene expression, and the prediction model that estimated gene expression with cis-SNPs was constructed (Zhou et al., 2013). Afterward, the prediction model was utilized to estimate the gene expression values of the 1,358 individuals without transcriptome sequencing data but with genotypes (Dai et al., 2019; Zhou et al., 2020). Finally, all of the gene expression data were integrated with phenotypes to implement TWAS with LMM:

y=Xb+Wu+e,

where *y* and *b* are the same as in GWAS, *W* is the design matrix of the gene expression matrix, which is constructed with transcripts per kilobase million (TPM) (Luningham et al., 2020), u is the vector of gene effect, e is the random residual, and $e∼N\left(0,\;I\sigma_{e}^{2}\right)$ . In TWAS, the significant gene test was implemented with FDR, and the threshold of the *p-*value was set at *FDR*×*n*/*m*, where FDR = 0.01, n is the number of genes with a *p-*value < 0.01, and m is the total number of genes in the LMM (Benjamini and Hochberg, 1995).

Fisher’s combined test (FCT): The *p*-value in GWAS of each SNP in the top 10% of most associated SNPs was assigned to the nearest gene and then combined with the *p*-value in TWAS (linear model with multi-dimensional scaling (MDS) principal coordinates + 5 probabilistic estimation of expression residuals (PEERs)) for that same gene using Fisher’s combined test as implemented in the sumlog method in the metap package (Dewey 2017) in R. TWAS *p*-values for genes which were not tested in TWAS was set to *p* = 1 prior to combining with GWAS *p*-values (Kremling et al., 2019). Similarly, the significant gene test was implemented in FCT using FDR with an identical threshold of the *p-*value.

Gene functional analysis: Gene Ontology (GO) is a database describing the function of genes and proteins. It annotated the genes into three types of terms: MF, BP, and CC (Ashburner et al., 2000). The KEGG database integrated the genome, regulatory network, and system function information (Kanehisa et al., 2016). To explore the function of candidate genes, we applied DAVID (https://david.ncifcrf.gov/) to implement GO and KEGG analyses of the genes and constructed the associated network of the gene-participated terms using ToppCluster (https://toppcluster.cchmc.org/).

## Results

### Genome-wide association studies

**FIGURE 1:**
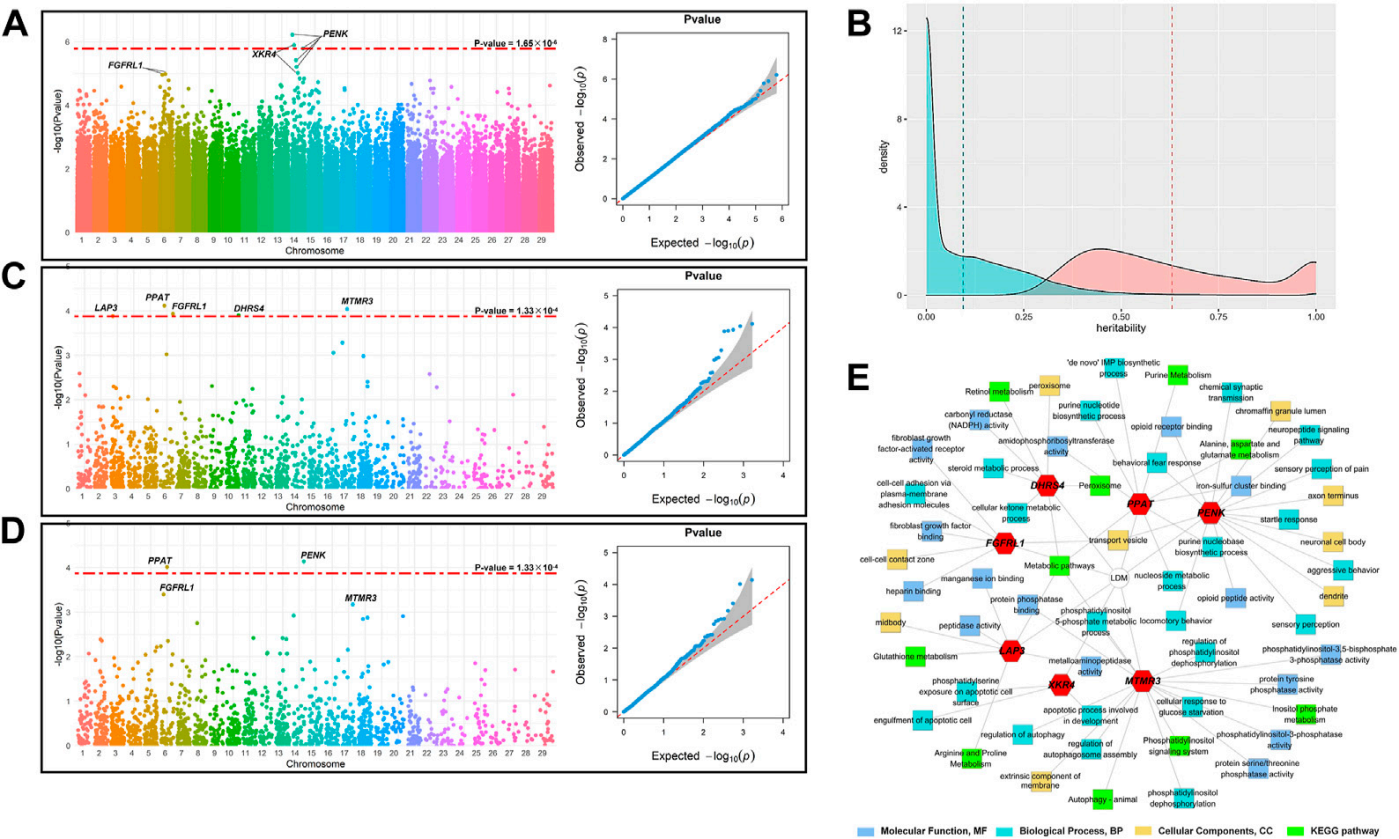
Identification of the candidate genes for LDM. **(A)** Manhattan plot and QQ plot of GWAS; the red dashed line indicates the threshold of Bonferroni’s multiple test, *p* = 1.65 × 10^−6^. **(B)** Manhattan and QQ plots of TWAS; the red dashed line indicates the threshold of the corrected *p*-value with FDR = 0.01 (*p* = 1.33 × 10^−4^). **(C)** Manhattan and QQ plots of FCT; the red dashed line indicates the threshold of the corrected *p*-value with FDR = 0.01 (*p* = 1.33 × 10^−4^). **(D)** Distribution of the estimated heritability of the genes. The blue area represents the distribution of the heritability of all gene expression, and the blue dashed line represents the mean of the heritability estimates of converged gene expression (0.152 ± 0.263); the orange area represents the expression of 1,650 significant genes (*p* < 0.05), and the orange dashed line represents the mean of the heritability estimates of significant gene expression (0.631 ± 0.324). **(E)** Results of GO and KEGG analyses of the candidate genes.

Figure 1A shows the Manhattan plot and QQ-plot of the GWAS analysis of LDM. The QQ-plot showed that there was no apparent systematic deviation. Most of the points were distributed around the diagonal (the expansion coefficient is 1.05), which means that only a few SNPs were associated with the phenotype. The threshold of the *p-*value (*p* = 1.65 × 10^−6^) was set with Bonferroni’s multiple test, and three SNPs in the 14th chromosome were significantly associated with the phenotypes, among which BovineHD1400006836 and BovineHD4100011289 were annotated to *PENK*, BTB-00557532 was annotated to *XKR4*, and the reference cattle genome was ARS-UCD1.2 more details are demonstrated in Table 1.

**TABLE 1:** Details of the significantly associated SNPs identified by GWAS.

SNP	Chromosome	Location^a^	MAF^b^	Length^c^	Candidate gene^d^	*p-*value^e^
BovineHD1400006836	14	23,552,180	0.35	5,312	*PENK*	6.09E-07
BTB-00557532	14	24,643,266	0.38	32,311	*XKR4*	1.26E-06
BovineHD4100011289	14	23,553,712	0.22	6,844	*PENK*	1.63E-06

^a^ The SNP position (bp) on ARS-UCD1.2.

^b^ The minor allele frequency.

^c^ The distance between SNP and the nearest gene.

^d^ The nearest genes found on the Ensemble database (www.ensembl.org).

^e^ *p-*values calculated by LMM.

### Transcriptome-wide association studies

After removing the genes with the average TPM (transcripts per kilobase million) less than 0.1, the expression levels of 15,325 genes of 120 individuals were assigned as phenotypes and 15,401 cis-SNPs, located within 1 Mb of the physical position of the gene, were assigned as the genotypes, and the heritability of the gene expression was estimated with REML. As shown in Figure 1B, the heritability of 15,324 genes converged in the progress of REML, and the average heritability was 0.152 ± 0.263. With *p* < 0.05 as the threshold, 1,650 genes were retained for the subsequent analysis, with an average heritability of 0.631 ± 0.324.

The Manhattan plot and QQ plot of TWAS for LDM were demonstrated in Figure 1C. The QQ-plot indicated that most points were distributed around the diagonal (expansion coefficient *λ* = 1.03), and several genes were significantly associated with LDM. After being corrected for the false discovery rate (FDR) of 0.01, the threshold of the *p-*value was set at 1.33 × 10^−4^, and five genes were found to be significantly associated with LDM. The location of these genes is listed in Table 2. *PPAT* (*p* = 7.68 × 10^−5^), *MTMR3* (*p* = 9.11 × 10^−5^), *FGFRL1* (*p* = 1.17 × 10^−4^), *DHRS4* (*p* = 1.26 × 10^−4^), and *LAP3* (*p* = 1.32 × 10^−4^) were located in chromosomes 6, 17, 6, 10, and 3, respectively.

**TABLE 2:** Details of the five candidate genes identified by TWAS.

Gene	Chr	Start^a^	End^a^	Effect ± SD^b^	*p-*value^c^	h^2^ ± SD^d^
*PPAT*	6	71,782,614	71,821,764	-0.0031 ± 0.00089	7.68E-05	0.76 ± 0.32
*MTMR3*	17	68,971,211	69,102,722	-0.0060 ± 0.00020	9.11E-05	0.61 ± 0.31
*FGFRL1*	6	117,346,407	117,358,800	0.0020 ± 0.00068	1.17E-04	0.58 ± 0.30
*DHRS4*	10	21,088,232	21,100,627	-0.0069 ± 0.0022	1.26E-04	0.68 ± 0.32
*LAP3*	3	37,140,752	37,166,191	0.018 ± 0.0031	1.32E-04	0.70 ± 0.34

^**a**^ The SNP position (bp) on ARS-UCD1.2.

^**b**^ The effects of gene expression calculated by LMM in TWAS.

^**c**^ *p-*values calculated by LMM.

^**d**^ The heritability of gene expression calculated by REML.

### Fisher’s combined test

The Manhattan plot and QQ plot of FCT analysis are shown in Figure 1D. The expansion coefficient *λ* of the QQ-plot was 1.02 with no systematic deviation, and most points were distributed on the diagonal, with only a minority of points floating above the diagonal. As with TWAS, the threshold of the *p*-value was set at 1.33 × 10^−4^. The Manhattan plot indicated that FCT identified two candidate genes significantly associated with LDM, namely, *PPAT* (*p* = 9.69 × 10^−5^) and *PENK* (*p* = 7.26 × 10^−5^), which were also identified by TWAS and GWAS, respectively.

### Functional analysis of candidate genes

Combining the results of GWAS, TWAS, and FCT, *PENK*, *XKR4*, *PPAT*, *MTMR3*, *FGFRL1*, *DHRS4*, and *LAP3* were identified as the candidate genes of LDM. To further explore the function of these genes, we performed GO and KEGG analyses of these genes. The results are demonstrated in Figure 1E. These candidate genes participated in 48 GO terms, which contained 16 molecular function (MF) terms, 23 biological progress (BP) terms, and nine cellular component (CC) terms. For MF, the candidate genes mainly function in the progress of fibroblast growth factor activity regulation (GO:0005007 and GO:0017134), NADPH activity (GO:0004090), and serine, threonine, and tyrosine metabolism (GO:0004722 and GO:0004725). KEGG pathway analysis found that candidate genes were involved in 10 pathways, mainly including amino acid and peptide metabolism, signal transduction pathway, purine metabolism, and other biological processes.

## Discussion

Abundant studies have proven that GWAS could precisely locate the candidate loci for the quantitative traits in livestock breeding, especially for the traits with high heritability. It was one of the most widespread methods used in plant and animal improvement programs. However, the regulatory mechanism from SNP to phenotypic variation was still unknown in most cases, and it was impossible to determine the genuine pathogenic gene of the trait associated with the candidate SNPs due to the linkage disequilibrium (LD) in the SNPs. In recent years, the innovation of sequencing technology provided more other omics biological information, transcriptome, metabolome, *etc*., and assisted in locating candidate genes more accurately. TWAS implement the association analysis based on the gene expression data with the phenotype to locate the candidate genes directly. The results of previous studies indicated that TWAS performed well in practice (Dai et al., 2019; Luningham et al., 2020; Li et al., 2021). In this study, we not only performed GWAS and TWAS individually but also utilized an ensemble approach, FCT, combining the results of GWAS and TWAS to locate the candidate genes for LDM.

For LDM in this study, we indented seven candidate genes by GWAS, TWAS, and FCT in total: *PENK* was located by GWAS and FCT, *PPAT* was located by TWAS and FCT, and the remaining five genes were only located by one of the methods. An et al. (2019) also located *PENK*, which was associated with the height of Brahman cattle and Nerol cattle populations. The studies on humans also found that *PENK* regulated cell development by encoding the opioid peptide growth factor (ORF) to affect height (Pryce et al., 2011). Zhan et al. (2014) found that a variation site (8p12.1) in *XKR4* was associated with human thyroid-stimulating hormone (TSH) secretion, and it was the candidate gene for the development traits in Brahman cattle, Korean yellow cattle, Chinese Holstein cattle, and Chinese Sujiang pig populations (Lindholm-Perry et al., 2012; Edea et al., 2020; Naserkheil et al., 2020; Xu et al., 2020). The protein encoded by *PPAT* was a member of the purine/pyrimidine phosphoribosyltransferase family, which was essential in regulating the proliferation, migration, and invasion of thyroid cancer. Gene function analysis found *PPAT* functionals in inosinic acid biosynthesis (GO:0006189), and GART was the functional partner of *PPAT*, which had a fundamental impact on nucleotide metabolism and internal environment balance (Welin et al., 2010). *MTMR3* is a member of the MTM family associated with muscular dysplasia, which participates in the cell progress of proliferation, differentiation, autophagy, and division by regulating the synthesis of myotube (Hnia et al., 2012). The reported studies have confirmed that *MTMR3* was the virtually candidate gene in the Holstein population for the quantitative traits, such as milk fat rate, milk yield, and milk protein content (Pimentel et al., 2011). *FGFRL1* encoded fibroblast growth factor receptor one, which plays a crucial role in the progress of cell adhesion, embryonic slow muscle fiber development, and bone tissue formation (Amann et al., 2014; Niu et al., 2015; Yang et al., 2016). Bluteau et al. (2014) indicated a slight reduction in the whole bone of the *FGFRL1* gene knockout mice. The study on Holstein also identified *FGFRL1* as a candidate gene for development traits in the Holstein population (Zhang et al., 2017). *DHRS4* encodes NADP(H)-dependent retinol dehydrogenase/reductase. The study on pigs found that rs196958886, one of the SNPs of this gene, may induce the peroxisome proliferator-activated receptor alpha (PPARα) gene, affect the interaction between fatty acids and glucose metabolism, and ultimately affect the quality of pork (Hwang et al., 2017). *LAP3* encodes leucine aminopeptidase, which is functional in protein metabolism and growth (Yao et al., 2020). Substantial studies on cattle found that *LAP3* was a candidate gene that affects important production traits such as visceral organ weight, body size, and carcass traits (Setoguchi et al., 2009; Bongiorni et al., 2012; Xia et al., 2017; An et al., 2018; An et al., 2020). Zheng et al. (2011) implemented association analyses between *LAP3* and milking traits in the Holstein population and concluded that *LAP3* was the vital candidate gene for milking traits.

## Conclusion

In conclusion, we identified seven candidate genes of LDM by GWAS, TWAS, and FCT based on genome and transcriptome information. According to the previous relevant studies and the results of gene function analysis, the candidate genes were mainly functional in the progress of the development of the body and the growth of muscle cells. Combining advanced breeding techniques such as gene editing with our study will significantly accelerate the genetic improvement of Huaxi cattle.

## Data Availability

The data presented in the study are deposited in the NCBI repository (https://www.ncbi.nlm.nih.gov/), accession number PRJNA721166, and DRYAD repository (https://datadryad.org/stash), accession number 10.5061/dryad.4qc06.
